# Massachusetts Veterinary Association

**Published:** 1891-07

**Authors:** Austin Peters

**Affiliations:** Secretary


*Massachusetts Veterinary Association.*—The regular meeting of the Massachusetts Veterinary Association was held at 19 Boylston Place, Boston, Wednesday evening, May 27, 1891.

President L. H. Howard in the chair. Members present: Drs. Billings, Blackwood, Emerson, Hadcock, Hitchings, Howard, Lee, Skally, Winchester, Winslow, and the Secretary. Honorary member, Dr. Stickney. Visitors: Dr. F. M. Perry and Mr. Andrew Ward.

The minutes of the previous meeting were read and accepted.

Unfinished business: The Secretary was instructed to look up the attendance of members during the past year, in order to decide who is to receive the rasp offered as a prize by Dr. Lee, for the best attendance. New business: Dr. Winchester suggests that the by-law requiring a thesis from candidates for membership be rendered inoperative for three months. It was found that in order to do this the Constitution would have to be amended. Dr. Winchester thereupon withdraws his suggestion. Motion made by Dr. Winchester that the Secretary notify all veterinary graduates in Massachusetts of the existence of the Association and invite them to join. Seconded by Dr. Winslow. Carried. Moved by Dr. Winchester, that two hundred copies of the Constitution be printed. Seconded by Dr. Winslow, and carried.

Papers and discussions: Dr. Billings was called upon by the President to address the Association. Dr. Billings delivered an address, a synopsis of which is given, as follows:

He prefaced his remarks by saying that he felt he was among his friends, and that he would have renounced his title of veterinarian long ago if it were not for the fact that he was a member of the Massachusetts Veterinary Association. He then spoke briefly of Clark University, at Worcester, and said that some of his friends there would be happy to make a scientific investigation of the spinal cord of the horse which Dr. Howard had sent to Ward's Wharf, for the members of the Association to examine, the animal having an exaggerated form of stringhalt.

He then proceeded with the main topic of his discourse, saying that the Massachusetts Veterinary Association must do all in its power to push the profession forward in this State. If we put our shoulders to the wheel, he thinks we can accomplish a great deal for the advancement of our profession. One way to do this was to secure provision from the next Legislature to appoint a scientific veterinarian, at the State Experiment Station, at Amherst, for the investigation of the infectious animal diseases. He thought that the State ought to be willing to appropriate $15,000 for this work. What we want to do is to start this work at Amherst and show the people that the veterinary profession amounts to something. In Massachusetts we may not have exactly the same conditions as in the West, but we have work here to do, if we can only get a start, and make a centre at Amherst, with the profession back of it. If we only had a place in Massachusetts where we could refer our doubtful pathological questions, it would help to advance our profession more than anything else. What we want is to have our profession send in a strong petition to the Legislature to get a place established, a place where we can put our best man, and out of which good pathological work can come.

After Dr. Billings closed his remarks, the following discussion ensued:

Dr. Lee thought that one way to form a veterinary centre might be to raise funds to pay the expenses of a farm, to which we might send our unusual surgical cases, or surgical cases which the owners did not care to see through to the end; the members of the Association to send the cases and render their services free.

Dr. Winchester thought that Dr. Billings had struck the right keynote in speaking of Amherst. He also referred to Dr. Goessmann's work on feeding experiments with animals, and said he had obtained results that would astonish the world when he was ready to publish them. Dr. Page is a good man, although without the special training required by a scientist. Dr. Humphrey is a good microscopist, and with such a chemist as Dr. Goessmann, and with the buildings already built, there is a valuable nucleus ready for immediate work.

The President said that Dr. Billings had warmed us up on a new tack, but that we are not very good as politicians to get such work done. As an illustration of how useful a veterinary department at the Experiment Station at Amherst would be to the profession, and to owners of animals, Dr. Winchester cited an incident in his own practice. A farmer lost several cattle, and was afraid that he had some contagious disease among them, or that some malicious person was poisoning them. Dr. Winchester sent some of the viscera from animals which had died to Prof. Goessmann, who found that the trouble was due to acetic poisoning as a result of feeding too much ensilage. The food was changed, and the survivors recovered.

Dr. Billings mentioned a disease in horses, fed upon ensilage, resembling cerebo-spinal meningitis, which was undoubtedly due to some germ formed during the fermentation of the food.

Dr. Peters asked Dr. Billings if he thought Clark University would establish a department for the investigation of infectious animal diseases, in case we failed to secure anything at Amherst. Dr. Billings thought that Clark University is a great thing, and said that Dr. Hall is willing to do anything he can to help him, but that he favored Amherst *as the place*.

Dr. Stickney thought that the work done at Amherst in feeding animals, and vegetable physiology, was about the only systematic work of any importance done in this country, with the exception, perhaps, of a little work on rabies and tuberculosis, which had been done at the Harvard Medical School.

The conversation then turned to a discussion of Dr. Howard's case of stringhalt at Ward's Wharf.

D. Winchester made the motion that the Secretary be instructed to write to Clark University, asking if they would like Dr. Howard's stringhalt case for purposes of autopsy, and if they would that they be furnished with the result of both microscopic and macroscopic examination, and that the bones of the hind legs from the tibias down be returned to the Association.

Seconded by Dr. Winslow. Carried.

Dr. Winchester spoke of a case in his practice at Lawrence, of a dog bitten by another one last July, which showed no evidences of rabies until the other day. Dog upon post-mortem, by Dr. Winchester, was found to have foreign bodies in stomach, stomach congested, brain congested, and effusion in the ventricles. He had bitten other dogs. Query: Will they go ten months before showing symptoms? Dr. Winchester then spoke of a dog which had not been in contact with other dogs for twelve months. He showed rabiform symptoms, and was poisoned by him. On post-mortem the stomach showed erosions; brain not examined. Diagnosis in this case, gastritis.

Dr. Lee reported a case of hernia in a grey gelding. The animal was a young one which the owner had just bought, and the first night he was brought home kicked over the partition of the stall, the near hind leg hanging over the partition. After freeing him the owner found he had a large swelling above the left flank, but did not attach much importance to it. He did not seek professional advice, but took the opinion of a friend, who told him it would be a "gathering" and to wait a few days until it "softened," and then to "stick a knife in it." After a few days the owner "stuck a knife in it," and as nearly as Dr. Lee could find out a little blood-stained serum ran out. Later in the day the horse was taken with colicy pains, and Dr. Lee was sent for. He found a loop of intestine protruding from the opening made by the knife, and already becoming gangrenous. After getting Dr. Peters to see the case in consultation, it was decided that the only thing to do was to destroy the animal. Upon post-mortem a rent six inches long was found in the muscular coat of the abdomen, in the upper part of the flank, the peritoneum and intestines being retained by the skin and subcutaneous connective tissue. The animal had been regularly worked for a week or ten days after receiving the injury up to the day the owner had opened the swelling. The loop of protruding intestine was the size of a man's fist, strangulated at the opening in the skin, and had commenced to mortify.

Dr. Lee also reported a case of a horse that had had trouble with his teeth for four years. About three years ago the first upper molar tooth had been pulled out; and the animal's mouth afterward neglected, the unopposed lower molar grew until it had worn a hole in the palate, exposing the lower ends of the turbinated bones and causing them to ulcerate. There was a disagreeable-smelling discharge from the nostril and the horse made a roaring sound when put to any exertion. Dr. Lee removed the overgrown first lower molar, and knocked out two pieces of the root of the upper molar which remained, leaving a hole as large as a half dollar in the floor of the nostril. This hole had to be plugged with oakum when the horse drank, as if left open it prevented his making a vacuum with his mouth, and hence he could not drink. The hole is now closing rapidly with healthy granulation tissue. There are healthy granulations of the ends of the ulcerated turbinated bones, the discharge is ceasing, and has lost its disagreeable odor, and the horse now feeds easily, and is rapidly gaining in flesh and strength.

Dr. Winchester reported a case of fracture of the pubic symphysis in a mare which was trying to foal. She was standing upon a wet floor when her hind feet flew out sidewise, and the ischio-pubic symphysis split its entire length.

Dr. Winslow said he had a case of ulceration of the palate similar to the one reported by Dr. Lee.

Dr. Blackwood reported a case of rupture of the uterus of a mare used on the horse cars. She was in foal, and last winter fell down and the car ran on to her. She did not seem much hurt at the time, and was soon able to resume work again. One night this spring he was sent for in a hurry to see the mare, as she was trying to foal, and evidently required assistance. When he arrived, the mare was dead, and a quantity of large intestines protruded from the vagina. Upon post-mortem examination he found a rent at the neck of the uterus through which the intestines escaped, and a fully-developed foal in the uterus. The edges of the rent were fully cicatrized, showing the tear to be an old one, and in all probability the result of the fall last winter.

Dr. Howard said that Mr. Ward must often have interesting experiences, and called upon him for a few remarks.

Mr. Ward related a case that might have been mistaken for rabies by members of the laity, which occurred in one of his hounds. The dog acted strangely one morning when he came down stairs, snapped at him and the maid when they went to touch him and acted in a very unusual manner. He told his man he was afraid the dog was going mad, and to chain him securely in the stable until his return that evening. He then set out for his place of business, but had not gone far when he met a neighbor who told him that one of his hounds had been smelling around one of his (the neighbor's) bee hives that morning and had been pretty thoroughly stung. Mr. Ward said that the case of rabies was at once accounted for, and he told the man to unchain the dog as soon as he returned home, and the dog was perfectly well up to the present time. He thought that many cases of so-called rabies could often be accounted for in a similar way. Mr. Ward also kindly offered to keep the pony with stringhalt at his wharf as long as the Association wished it to remain there.

Dr. Winchester reported a case of fistula of Stenos' duct in a horse.

Dr. Winchester moved that the Association extend a vote of thanks to Mr. Ward for his courtesy and kindness in keeping the horse with stringhalt at his wharf for our observation.

Seconded by Dr. Stickney. Carried unanimously.

Meeting then adjourned.

AUSTIN PETERS,
*Secretary.*

